# Analysis of the physicochemical properties of antimicrobial compositions with zinc oxide nanoparticles

**DOI:** 10.1080/14686996.2019.1697617

**Published:** 2019-12-02

**Authors:** Jolanta Pulit-Prociak, Jarosław Chwastowski, Laura Bittencourt Rodrigues, Marcin Banach

**Affiliations:** Faculty of Chemical Engineering and Technology, Institute of Chemistry and Inorganic Technology, Cracow University of Technology, Cracow, Poland

**Keywords:** Zinc oxide nanoparticles, PVA, coatings, 306 Thin film / Coatings

## Abstract

In this study, an antimicrobial composition based on polyvinyl alcohol (PVA) and zinc oxide (ZnO) was developed. The aim of the work was to obtain a film-forming product for antimicrobial treatment of surfaces. To improve the physical, mechanical, and film-forming properties of the compositions, three natural stabilizing agents were added to the formulation: gelatine, guar gum and hydroxyethyl cellulose. Formulations with different concentrations of each stabilizer were tested, and the physicochemical properties of the obtained products were measured. The size of zinc oxide particles in obtained compositions varied from 232 to 692 nm. The compositions had a slight acidic nature. Their pH ranged from 6.84 to 6.99. The average density of products was equal to 1.37 × 10^3^ (kg/m^3^). It was confirmed that zinc oxide nanoparticles do not penetrate through a model dermal membrane which is a desired effect concerning their toxicity. The antimicrobial activity of the obtained compositions was assessed against *Aspergillus niger* strain. After 24 h of studying, the growth inhibition was in 71% greater than in reference material. After statistical analysis of the results, it was concluded in order to achieve the most desirable physicochemical and utilitarian properties, the concentrations of gelatine, guar gum and hydroxyethylcellulose should be equal to 0.5%, 0.03% and 0.055%, respectively.

## GRAPHICAL ABSTRACT

**Figure:**
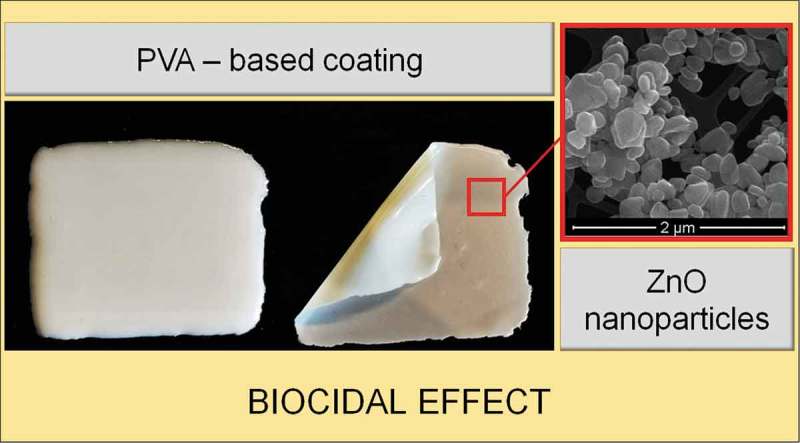

## 1. Introduction

Nanotechnology is a subject of growing and promising research in many fields such as in medicine, material science, pharmaceuticals, electronics, and semiconductors. Nanoparticles are defined by their size; they are particles between 1 and 100 nanometres, and they can lead to outstanding mechanical, optical, magnetic, thermal, biological, and chemical properties when compared to bulk materials. Due to their high surface area, nanomaterials present such properties even at low concentrations [1]. Among the metal oxide nanoparticles, zinc oxide (ZnO) is found in many applications because of its versatile properties. Nanoscale ZnO presents optical [2], semi- conductive [3], UV absorbing [4], and antimicrobial properties [5].

Bacterial and microbial infections are considered to be a serious threat to human health because of mutations and the resistance developed by these microorganisms through years of overexposure to antibiotics. Researchers are constantly working on the development of antimicrobial agents, and they often revisit known biocide components to study their mechanisms of action or to search for new ways to apply such substances. Owing to its microbicide properties, nano-ZnO can be used in food packing, textiles, cosmetics, personal care products, and biomedical applications [6,7]. Zinc oxide (ZnO) is a naturally occurring metal oxide found in mineral form as zincite. It is commonly used in industry for the production of rubbers, concrete, and paints [8]. Because of its electrical properties, it is widely studied in semiconductor research and development. In the pharmaceutical industry, ZnO is currently found in many applications such as in lotions, sunscreens, and other healthcare products [9]. According to the U.S. Food and Drug Administration (U.S. FDA) [10], zinc oxide is a Generally Recognized As Safe (GRAS) substance. Zinc oxide has a tendency to grow self-organized nanostructures. The unique and enhanced properties of ZnO nanoparticles (NPs) have opened more fields of study and applications for the material, such as in ceramics, pharmaceutical formulations, bioimaging and antimicrobial treatments. Similar to other nano-sized substances, nano-scaled ZnO presents new physicochemical, electrical, optical and structural properties, because the surface area/volume ratio is increased. Because of that, small quantities of ZnO NPs can present powerful properties when compared to the bulk material [9,11].

Gelatine (Gel.) is a natural hydrocolloid of animal origin. It is extracted through partial hydrolysis of collagen from skin and bones. It consists of a range of 18 amino acids that are bonded in an organized chain [12]. The amino acids in the gelatine structure are mainly glycine, proline, and hydroxyproline. This unique combination and arrangement is responsible for the helicoidal shape of the chain and for the gelling properties of gelatine [13,14]. Gelatine is the most commonly used thickening/gelling agent in many industries such as in food, cosmetics, and pharmaceuticals and in the medical field. This versatility is due to its unique characteristics such as good solubility in polar solvents, biodegradability, biocompatibility, and thermo-reversibility. When heated at above 338.15 K, gelatine is easily dissolved and incorporated into compositions. When cooled, the amino acid chains begin to form a triple-helix structure similar to collagen and this forms an elastic and firm hydrogel [14–16].

Guar gum (GG) is a polygalactomannan extracted from the endosperm of the Indian guar seeds *(Cyamopsis tetragonolobus)*. The seeds are mainly produced in India, but Pakistan and the USA are also big exporters. It is especially interesting for pharmaceutical and food industries because of its stabilizing, emulsifying, thickening and gelling properties, even at low concentrations. The chemical structure of guar gum consists of a (1-4)-linked β-d-mannopyranosyl backbone with randomly distributed (1-6)-linked α-d-galactopyranosyl branches [17]. Guar gum is a polysaccharide with high molecular weight, compared to other natural polymers, in the range of 1–2 × 10^3^/N_A_ kg. It presents high water solubility and water binding properties. When applied to gel compositions, guar gum contributes to the strength and elasticity of the gels [17,18].

Hydroxyethyl cellulose (HEC) is a water-soluble cellulose derivative. It is largely used as a thickening, suspending and stabilizing agent. HEC is obtained from the etherification reaction between cellulose and ethylene oxide [19]. Hydroxyethyl cellulose is applied in cosmetic, textile, paint, and food industries. It is gaining interest because of its non-ionic, non-toxic, water-retaining, solubility, and film-forming properties. HEC has been used to improve the rheological properties of suspensions, making them more elastic as the shear is increased [20,21].

Chitosan presents unique properties, many of them are of particular interest in the compositions prepared. Some of these properties are nontoxicity, biodegradability, bio-adhesion, and film-forming ability [22,23].

The applications of sucrose are not limited to its use as a sweetener in the food industry. It can be used as a natural plasticizer, stabilizer, and gelling agent, contributing to the rheological properties of the compositions [23,24]. It also has high viscosity, high solubility, and antioxidant properties [25].

Casein is a phosphoprotein derived from bovine milk. It is naturally assembled into micelles with a diameter of 50 to 300 nm (5 × 10^−8^ to 3 × 10^−7^ m). Its aggregates, or acid-casein, lose functionality and are insoluble. An alkali media is required to neutralize the pH and retrieve solubility and properties. In the case of neutralization with sodium hydroxide, the product is negatively charged and sodium caseinate is obtained [26]. Sodium caseinate is a common protein emulsifier in industry. In the present formulation, it is applied to improve the rheology and stability of the compositions [20].

Glycerol, or glycerine (propane-1,2,3-triol) is a sugar alcohol commonly found in nature either in its free form or in an ester form (glyceride). The hydroxyl groups present in the molecule give glycerol its hydrophilic and hygroscopic properties. Glycerol is a viscous, colourless, nontoxic, and stable component. In many industries, it is applied as an emollient, solvent, and humectant [27]. In the case of mixtures with hydrophilic polymers, as is the case of polyvinyl alcohol (PVA), glycerol acts as a plasticizer. It has an influence on the flexibility, toughness, elongation, and overall mechanical properties of the obtained films [28,29]. A composition formed between zinc oxide and PVA, where the metal oxide is dispersed in the polymer matrix, is the subject of this study. Polyvinyl alcohol is a polymer widely used in biomedical applications for its versatility, ability to form films, biodegradability, and biocompatibility. In order to obtain optimal film formation and properties, other polymers or proteins can be blended with PVA to act as thickening agents, plasticizers, and stabilizers [30]. The goal of this study was to develop and characterize a microbicide composition based on PVA and ZnO with the addition of natural stabilizing agents (gelatine, guar gum and hydroxyethyl cellulose). Secondly, it has the objective of estimating the effect of each individual stabilizing agent on the properties of the obtained products by performing statistical analysis on the physicochemical properties of the compositions.

## 2. Materials and methods

### 2.1. Materials

The following compounds were used in this study: poly(vinyl alcohol) (M = 72 kg/mol, ≥99.0%), chitosan (M = 100–300 kg/mol, high purity), gelatine (ACS reagent), guar gum (ACS reagent), glycerine (d = 1.26 × 10^3^ kg/m^3^, ACS reagent), 2-hydroxyethyl cellulose (M = 90 kg/mol, ACS reagent), sucrose (≥99.5%), casein (ACS reagent), zinc nitrate (≥99.9%), sodium hydroxide (≥98.0%), acetic acid (≥99.0%), sodium bicarbonate (≥99.7%), sodium chloride (≥99.0%), dipotassium hydrogen phosphate trihydrate (≥99.0%), magnesium chloride hexahydrate (≥99.0%), hydrochloric acid (≥37.0%), calcium chloride (≥93.0%), sodium sulfate (≥99.0%), and tris(hydroxymethyl)aminomethane (≥99.8%). The following compounds were used in the microbiological tests: peptone (ACS reagent), yeast extract (ACS reagent), ammonium sulfate (≥99.0%), magnesium sulfate (≥99.0%), monobasic potassium phosphate (≥99.0%), CaCl_2_ (≥99.0%), and saccharose (≥98.0%). All compounds were provided by Sigma Aldrich (Germany). The *Aspergillus niger* strains used in the study were taken from the American Type Culture Collection (ATCC). All compounds were provided by Sigma Aldrich (Germany). All solutions were prepared with deionised water (Polwater, 1.8 × 10^−7^ S).

### 2.2. Methods

#### 2.2.1. Obtaining zinc oxide nanoparticles

In the present study, the zinc oxide nanoparticles were obtained according to the precipitation technique described by Pulit-Prociak and Banach [31,32]. The reaction occurs in two steps. The first part of the process consists of the reaction between sodium hydroxide and the zinc precursor, forming a suspension of precipitated zinc hydroxide and sodium nitrate. The next step occurs in a high pressure reactor, as a way to accelerate the dehydration of zinc hydroxide. Briefly, aqueous solutions of zinc nitrate (Zn(NO_3_)_2_) at a concentration of 1 × 10^3^ mol/m^3^ and sodium hydroxide (NaOH) at a concentration of 2 × 10^3^ mol/m^3^ were prepared. 1.8 × 10^−4^ m^3^ of each solution were then mixed together manually at room temperature. This resulted in the mixture gelling, which indicated the formation of zinc hydroxide (Zn(OH)_2_) precipitate. The following step of the reaction consisted in the dehydration of the precipitate under high pressure and temperature. The reaction occurred at 453.15 K and 6 × 10^5^ kg/m·s^2^ for 1.8 × 10^3^ s in a laboratory pressure reactor. The resulting product was then filtered by gravity to separate water and ZnO. After filtering, the product was washed to remove any residual sodium nitrate and then dried at 378.15 K for 8.64 × 10^4^ s. The dry product was manually ground to a fine white powder. The resulting product was then analysed by X-ray diffraction (XRD) (X’Pert PW 1752/00, Philips, The Netherlands). Fourier-transform infrared spectroscopy (FTIR) measurements were carried out in the range 400–4000 cm^−1^, on powders, using a Nicolet 380 spectrophotometer (Thermo Fisher, USA). The surface morphology and composition of ZnO nanoparticles was investigated by scanning electron microscopy (SEM; 1430 VP microscope, LEO Electron Microscopy, UK) combined with energy-dispersive X-ray spectroscopy (EDX).

#### 2.2.2. Obtaining compositions with zinc oxide nanoparticles

Table 1 shows the ingredients of the compositions.

**Table 1.** Ingredients of the compositions.

Component	Concentration in the product (%)
PVA	7.00
Sucrose	0.12
Hydroxyethyl cellulose	0.010; 0.055 or 0.100
Guar gum	0.01; 0.03 or 0.05
Gelatine	0.1; 0.3 or 0.5
Chitosan	0.01
Glycerol	14.00
ZnO nanoparticles	3.00
Casein	0.40
Water	up to 100

All prepared compositions had the same base formulation but different concentrations of the three stabilizing agents: gelatine, guar gum, and hydroxyethyl cellulose. When the percentage content of cellulose, gum or gelatine changes, the percentage content of the remaining ingredients remains the same. The study was planned based on a design of experiments (DOE) according to a central composite plan. It resulted in obtaining compositions which varied in the concentration of the stabilizing agents (Table 2).

**Table 2.** DOE for the stabilizing agent concentrations and measured properties.

Sample	Input parameters			Output parameters			
	Concentration in the product (%)			pH	Density × 10^3^ (kg/m^3^)	Viscosity (kg/m·s)	ZnO nanoparticles size (nm)
	Gelatine	Guar Gum	Hydroxyethyl cellulose				
L1	0.100	0.010	0.010	6.91	1.324	0.364	232
L2	0.100	0.010	0.100	6.92	1.385	0.412	279
L3	0.100	0.050	0.010	6.88	1.368	0.630	307
L4	0.100	0.050	0.100	6.91	1.422	0.369	315
L5	0.500	0.010	0.010	6.87	1.410	0.262	381
L6	0.500	0.010	0.100	6.97	1.319	0.321	411
L7	0.500	0.050	0.010	6.92	1.400	0.358	417
L8	0.500	0.050	0.100	6.92	1.300	0.461	446
L9	0.100	0.030	0.055	6.96	1.362	0.448	519
L10	0.500	0.030	0.055	6.93	1.373	0.556	528
L11	0.300	0.010	0.055	6.99	1.332	0.453	573
L12	0.300	0.050	0.055	6.91	1.417	0.439	580
L13	0.300	0.030	0.010	6.84	1.376	0.341	617
L14	0.300	0.030	0.100	6.94	1.356	0.350	625
L15	0.300	0.030	0.055	6.90	1.389	0.395	692
Ref.	0.300	0.030	0.055	5.43	1.368	0.290	-

A blank sample with no ZnO nanoparticles was also prepared as the reference material.

Firstly, aqueous solutions were obtained for almost all of the components. Only chitosan was dissolved in acetic acid solution (at a concentration of 1.5% v/v), and casein was dissolved in a solution of NaOH (at a concentration of 3 × 10^2^ mol/m^3^). After preparing the needed solutions, the compositions were obtained. Initially, PVA was added to the container followed by deionized water. The polymer was stirred until it was completely dissolved in water and foaming of the mixture was observed. Then, the other reagents were added one by one, according to the order provided in Table 2. All of the components were placed in plastic containers with 1 × 10^−4^ m^3^ capacity and kept in a water bath at 336.15 K where they were stirred at 500 rpm (8.33 Hz). The concentrations and volumes of the starting solutions of the ingredients were calculated to obtain the final compositions of each reagent, and the concentration values are provided in Table 2. After the addition of the last ingredient (casein solution), the mixture was stirred for another 600 s to ensure good dispersion. Forty grams of the final product were obtained for each sample.

Physicochemical, morphological, mechanical, and antimicrobial properties of the compositions were measured by different analytical methods. Density was calculated by a weighing method and pH was measured with an Elmetron (Poland) pH-electrode. Viscosity was tested in a Brookfield (USA) rheometer, model R/S Plus, mounted with a 25-2 cone and set to a shear rate of 1 × 10^2^ 1/s. The size of the ZnO nanoparticles in the obtained compositions was determined through Dynamic light scattering (DLS; Malvern Zetasizer, UK). For this purpose, the samples were prepared by dispersing 5 × 10^−8^ m^3^ of the composition in 1 × 10^−5^ m^3^ of distilled water and then mixing in an ultrasonic homogenizer (Hielscher UP50H, Germany) for 60 s.

For the intended application of the compositions, it is to be poured on surfaces contaminated with microorganisms. After a few hours, it will form a solid film, which can then be peeled from the surface. The mechanical properties of the films were determined in order to measure the force necessary to detach them from a polypropylene surface. An established volume of each sample (in liquid state) was poured onto a polypropylene surface of known area (1 × 10^−6^ m^3^/1 × 10^−4^ m^2^). The structure was left to dry and solidify for 8.64 × 10^4^ s, then the mechanical analysis was carried out. A Zwick 1445 testing machine (Zwick DmbH & Co.KG, Ulm, Germany) was used to measure the bond strength, a measure of the maximum and average force (kg/m·s^2^) required for peeling off the film. The elongation of the film (m) was also measured. The speed of the test was set to 2 × 10^−2^ m/60 s.

The FTIR technique was applied for the detection of typical ZnO vibrations and for the identification of various functional groups and chemical structures in the obtained compositions. Solid samples were scanned in a Nicolet 380 spectrophotometer (Thermo Fisher, USA) over the range of 400–4000 cm^−1^. The structure of the films containing ZnO nanoparticles was analysed by XRD and SEM-EDX.

##### 2.2.2.1. Transdermal diffusion

**Figure 1.**
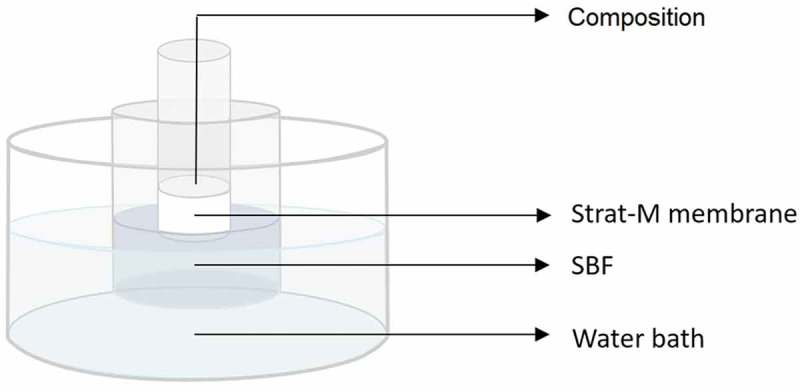
System for transdermal diffusion analysis.

A system for estimating the diffusion of Zn from the compositions through a synthetic membrane into simulated human skin (Strat-M, Merck, Germany) is presented in Figure 1. The obtained compositions are intended to be used as products which may have a contact with human body. The model dermal membrane is a synthetic, non-animal based model for transdermal diffusion testing that is predictive of diffusion in human skin. According to material safety data sheet this membrane is designed i.a. for screening of pesticides and chemicals. The fact that humans may be in contact with the applied composition was the reason for performing such studies. Simulated body fluid (SBF) served as the acceptor fluid. A transdermal diffusion membrane was attached to the end of a cylinder. The composition (1.5 g) was placed inside the cylinder in contact with the membrane. The membrane was positioned in a container with 2 × 10^−5^ m^3^ of SBF in such a way that it would be in contact with the surface of acceptor fluid but not submerged. The system was kept under magnetic stirring (400 rpm, 6.67 Hz) and inside a water bath at 309.15 K for 3600 s. After the contact time, the SBF was filtered through a paper filter (2 × 10^−7^ m) and the concentration of zinc was analysed by the inductively coupled plasma optical emission spectrometry (ICP-OES, Perkin Elmer, USA).

##### 2.2.2.2. Microbiological analysis

The antimicrobial properties of the compositions were tested against *Aspergillus niger. Aspergillus niger* as a model fungi organism and a tinea-causing pathogen is used in various microbial analyses. The compositions in this work can be used near swimming pools, where people come in contact with different fungi species. That fact was the reason for choosing this kind of organism. The analysis was performed according to the standard no. PN-EN 1275. The liquid samples (5 × 10^−7^ m^3^) were added to agar plates, then mixed with 2.5 × 10^−8^ m^3^ of the microorganisms in solution. The concentration of cells was equal to 1.5 × 10^7^ cfu/ml (1.5 × 10^13^ cfu/m^3^). A control sample, with no addition of the antimicrobial composition, was prepared to observe the growth of microorganisms. The growth inhibition of the fungi in the samples containing the samples was observed after 8.64 × 10^4^ s and 1.728 × 10^5^ s of incubation.

##### 2.2.2.3. Statistical analysis

Statistical analysis was performed based on one-way analysis of variance (ANOVA). The significance of the differences was evaluated through a test-F. The value of p < 0.05 was established as significant in all cases. The utility function profiles, with respect to certain independent parameters, were prepared. Through these profiles, it was possible to determine the changes in the dependent parameters when changing the values of the independent parameters. Approximation profiles were obtained, informing which specific values of input parameters ensure the most desirable (useful) estimated output factors. Approximated values are converted into a utility scale which includes the values of 0 (undesired effects) to 1 (desired effects). From the approximation profiles obtained, it was possible to optimize the usability of the compositions.

## 3. Results

### 3.1. Zinc oxide nanoparticles

**Figure 2.**
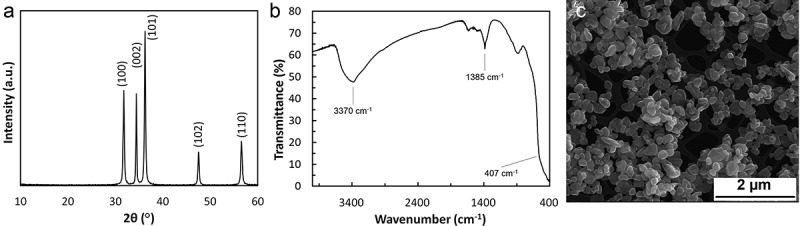
(a) XRD of prepared ZnO nanoparticles; (b) FTIR spectra of prepared ZnO nanoparticles; (c) SEM microphotographs of prepared ZnO nanoparticles.

A fine white powder of zinc oxide nanoparticles was obtained. Figure 2(a) presents the X-ray diffraction pattern of the produced material. Based on this, the phase and crystal parameters may be calculated. The strong intensity and good sharpness indicate that the obtained zinc oxide was well crystallized. The highest peak which appears at 36.23° of 2θ is obtained along (101) orientation. The observed peaks are located at 2θ = 31.74°, 34.40°, 36.23°, 47.52° and 56.56°. This pattern matches with the lattice planes (100), (002), (101), (102) and (110) respectively. These XRD results are characteristic for pure zinc oxide [33] and are in line with the standard card of ZnO powder sample [34]. The size of zinc oxide crystals was calculated based on the Debye-Scherrer equation: *d* = (Kλ)/(β·cosθ) in which K is the dimensionless shape factor (K = 0.94), λ is the X-ray wavelength (λ = 0.1540598 nm), β is the full width of peak at half maximum of the diffraction peak, and θ the Bragg angle. The calculation resulted in crystal size of 60 nm (6 × 10^−8^ m).

The bond between a metal and oxygen is usually indicated by absorption bands in the region below 1000 cm^−1^. They result from inter-atomic vibrations [35]. The stretching mode of Zn-O was confirmed by an absorption band which occurs between 400 and 590 cm^−1^. The band at around 1400 cm^−1^ may be assigned to H–O–H bending vibration mode due to the adsorption of moisture. Also, a wide band between 3000 and 3650 cm^−1^ indicates the presence of water (Figure 2(b)).

The shape and size of obtained zinc oxide particles were assessed by SEM. The results shown in Figure 2(c) indicate the appearance of flattened balls or a rod like morphology of zinc oxide. The SEM microphotographs indicated that the diameter of nanoparticles is between 150 and 500 nm (1.5 and 5 × 10^−7^ m). The particles were distributed homogenously on all analysed surfaces.

### 3.2. Compositions with zinc oxide nanoparticles

**Figure 3.**
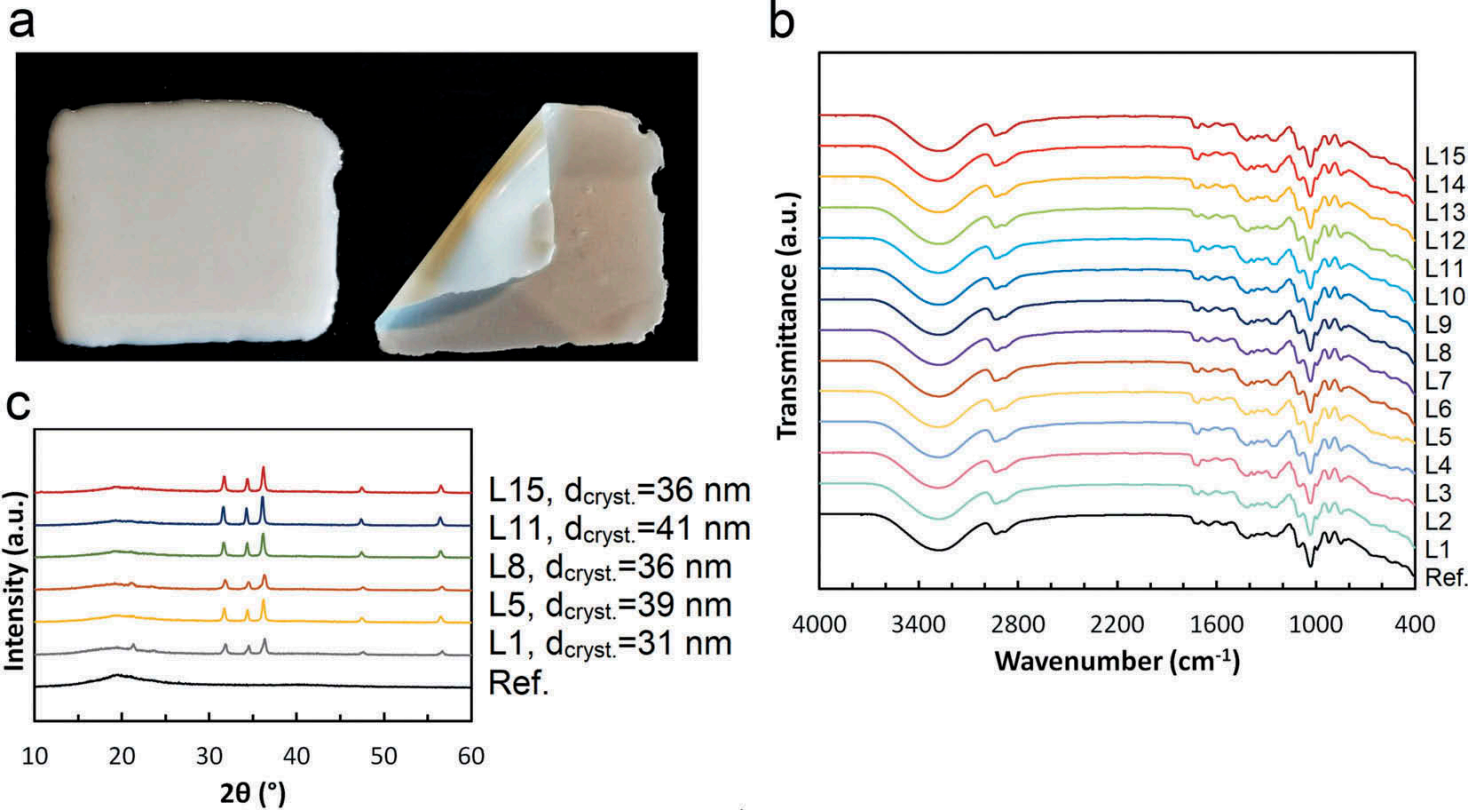
(a) Prepared composition before and after film formation; (b) XRD of prepared compositions; (c) FTIR spectra of prepared compositions.

The obtained compositions resulted in white, well dispersed suspensions. After some time, phase separation was observed and this required homogenizing the composition before every analysis. Figure 3(a) shows the composition applied to a surface in its liquid form (left hand side) and the film formed after 24 h (right hand side).

The results from pH, density, and viscosity measurements are presented in Table 2. It can be observed that the pH of the reference sample is lower than the rest, and it is equal to 5.43. The pH values of the prepared compositions are higher and vary from 6.84 to 6.99, which is not a significant difference. The higher pH values of obtained compositions result from the presence of zinc oxide nanoparticles. The density of obtained compositions varied from 1.300 × 10^3^ to 1.422 × 10^3^ kg/m^3^, and the density of reference sample was included within this range. The viscosity values were from 0.262 to 0.630 kg/m·s. The reference sample had low viscosity, but its value was included in this range. The liquids were dense and viscous enough to be applied onto a specific area without leaking, but at the same time they spread easily on the surface. The film was observed to be completely solidified 8.64 × 10^4^ s after the application, and it was easily peeled from the surface without breaking or requiring excessive strength.

It may be noted that after incorporation of zinc oxide nanoparticles into the compositions, their size enlarged. The particles varied from 232 to 692 nm (Table 2), and this suggests that agglomeration of the zinc oxide nanoparticles occurred. The prepared pure zinc oxide nanoparticles were not stabilized by any stabilizing agent, and this could be the reason of their aggregation into bigger agglomerates.

The FTIR analysis was performed to identify peaks related to the presence of characteristic groups in the compositions. Figure 3(b) presents the obtained spectra. One may note the similarity between positions of peaks and their intensity in all samples, indicating a good homogeneity through the compositions. Absorption peaks in the region of 470 cm^−1^ are characteristic of the Zn-O bond. A Zn-OH band at wavenumber 860 cm^−1^ is also present in all samples, and it is related to the water present in the composition, which is also indicated by the stretching bond between 3000 and 3600 cm^−1^ characteristic for O-H [31,36]. Other bands at around 2920 cm^−1^ and 2670 cm^−1^ are related to the symmetrical and asymmetrical C-H tensile vibrations from the aliphatic chains and terminal CH_3_ groups which are present in organic matter. The bands at wavenumbers 1733 cm^−1^ and 1640 cm^−1^ correspond to C = O and C-O stretching vibrations, respectively. The characteristic bands at 1400 cm^−1^ and 1367 cm^−1^ are observed for the bending vibrations of CH_2_ and COH. The band at 1070 cm^−1^ is related to C-O-C bending vibrations, and the bands at 1030 cm^−1^ and 990 cm^−1^ result from C-H and C-C stretching vibrations. The oscillation of the OH group is indicated by the band at 916 cm^−1^. The band at wavenumber 840 cm^−1^ results from polysaccharide rings. Based on the absorption spectra, a high glucose and poly(vinyl alcohol) content in the compositions may be confirmed. The spectra obtained for both tested compositions and the reference material are similar, which confirms the identity of the matrix.

The obtained diffractograms are presented in Figure 3(c). XRD analysis indicates the occurrence of zinc oxide in all compositions. Peaks detected at the 2θ angle regions of 32°, 34°, 36°, 47° and 56° match the lattice planes (100), (002), (101), (102) and (110) respectively, which are characteristic for pure zinc oxide [30,37]. The intensity of the peaks is related to the high crystallinity of ZnO particles. Due to the lack of zinc oxide in the reference sample, its presence was not confirmed. The average size of crystallites was calculated with the Debye-Scherrer equation as 26–41 nm.

**Figure 4.**
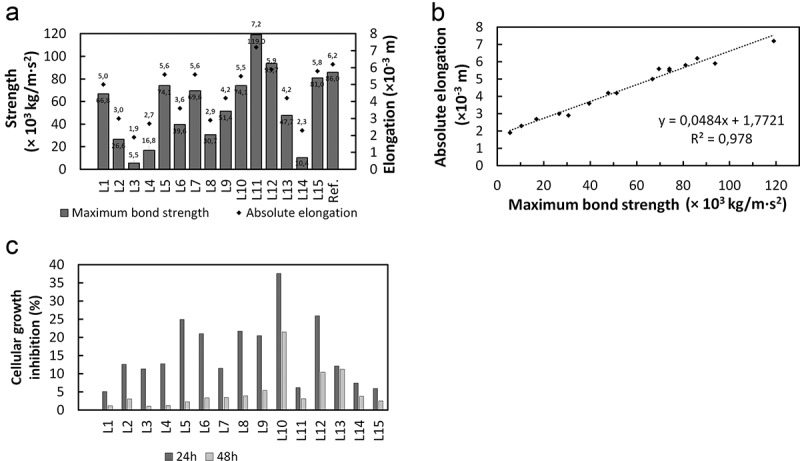
(a) Mechanical properties of the films; (b) Dependence of absolute elongation on maximum bond strength; (c) Cellular growth inhibition after 8.64 × 10^4^ s and 1.728 × 10^5^ s.

The mechanical analysis is based on measuring the adhesion strength between the solidified composition and a polypropylene surface. The results shown in Figure 4(a) indicate the force needed to tear off the solid film from the surface onto which it was applied as well as the elongation of the films. The force differed within the tested group. The highest value was achieved in Composition L11 which was formed with medium concentrations of gelatine and hydroxyethyl cellulose and with the lowest concentration of guar gum. The force which was needed to tear off the reference film did not stand out from the results obtained for the rest of the films. The influence of the input parameters on mechanical properties values will be discussed in the next section. Figure 4(b) presents the dependence of the absolute elongation of the tested films on the measured maximum bond strength of the films. The coefficient of determination (R^2^ = 0.978) suggests that the fit is very good, and when the maximum bond strength increases the absolute elongation of the films also increases.

**Figure 5.**
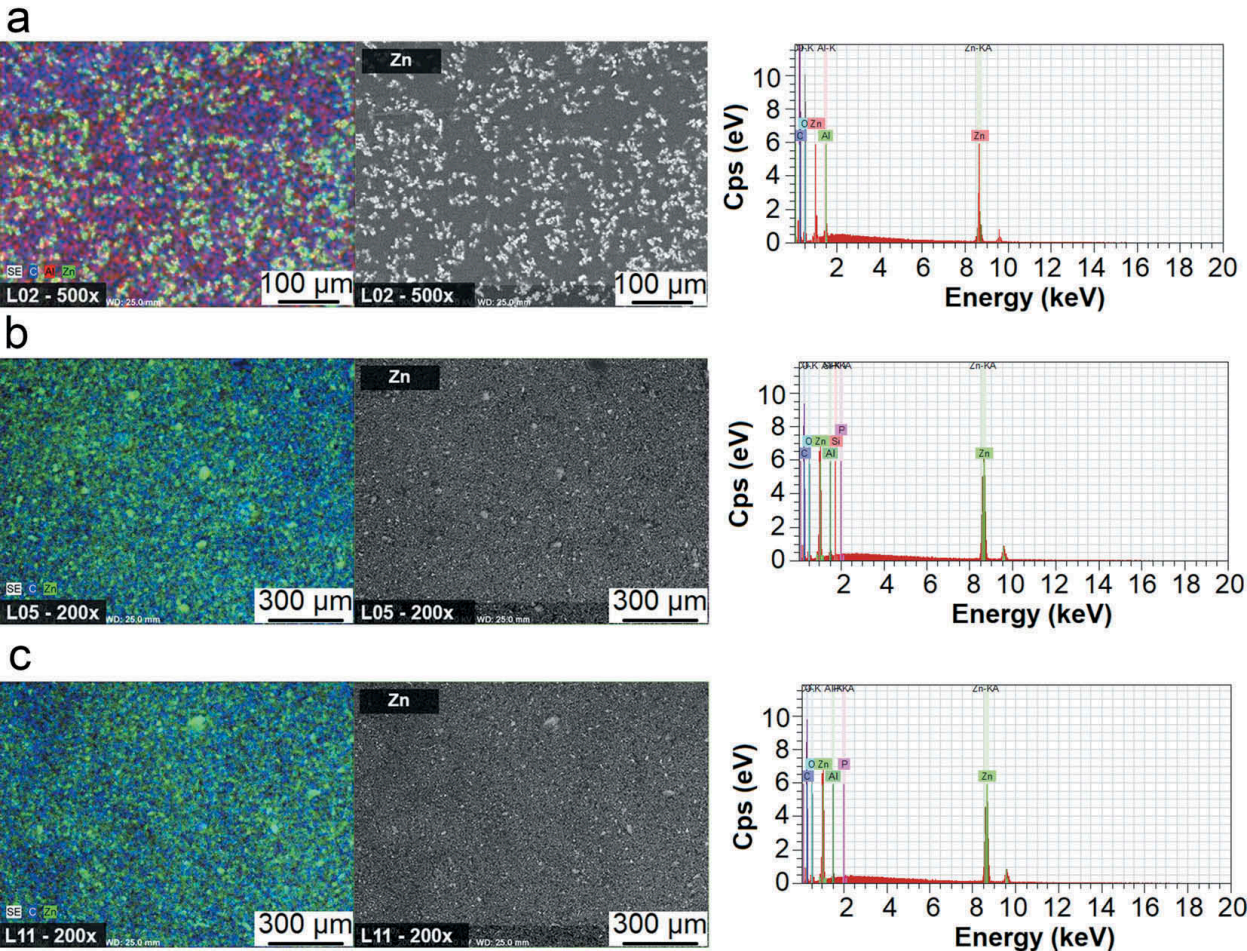
Elemental mapping, SEM microphotographs and EDX collective spectra of samples (a) – composition L2; (b) composition L5; (c) composition L11.

The distribution and nature of the particles present in the solid form of compositions were characterized by SEM-EDX. Figure 5 presents the obtained microphotographs. The images on the left present the main elements identified in the film, and in the middle is an isolation of the zinc detected, which originated from the ZnO nanoparticles. It can be noticed that the particles of ZnO were well dispersed and little agglomeration occurred. The shape and size of the particles are relatively homogeneous, without great variation in size.

An EDX collective spectrum was obtained as the response for bombarding the whole analysed area. The Zn peaks in all tested samples were intense, indicating the presence of ZnO particles through all of the analysed area. Carbon and oxygen are derived from the organic constituents of the formulation, and other detected elements can be related to instruments used in the preparation of compositions.

#### 3.2.1. Transdermal diffusion

The transdermal diffusion samples (acceptor fluids) were analysed by the ICP-OES technique. In all cases, the share of diffused zinc was below 1% or below the detection limit (compositions L3 – L8). In the 1.5000 × 10^−3^ kg of sample used for the analysis, 4.5 × 10^−5^ kg of ZnO was present. After the test, a maximum of 5.433 × 10^−^ kg/kg (composition L10) were transferred from the composition into the 2 × 10^−5^ m^3^ of SBF (acceptor fluid), which gives a total of 0.03% in mass of Zn diffused through the membrane. The low permeability through the membrane can be related to the size of the ZnO particles, which is above the traditional nanoscale range of 100 nm (1 × 10^−7^ m). Studies show that even much smaller ZnO nanoparticles, ranging from 30 nm to 160 nm (3 × 10^−8^ to 1.6 × 10^−7^ m), did not permeate through skin [38,39]. Another factor preventing the diffusion through the membrane is the use of stabilizing agents in the mixture. These create physical barriers around the nanoparticles and keep them suspended in the composition instead of migrating into the simulated body fluid.

#### 3.2.2. Microbiological analysis

The growth of microorganisms in the culture media was followed after 8.64 × 10^4^ s and 1.728 × 10^5^ s, and the inhibition of cellular growth against reference sample was calculated. Figure 4(c) presents the results. Despite the presence of organic compounds in the compositions, which could stimulate the growth of microorganisms, all samples presented antimicrobial activity, inhibiting the growth of *Aspergillus niger*. The results demonstrate the action of ZnO as a microbicide agent as well as the efficiency of compositions for the proposed application. The inhibition was stronger in the first 8.64 × 10^4^ s of exposure and particularly efficient in composition L10. Released zinc oxide nanoparticles are able to penetrate the cell membrane of microorganisms. Thus the lipids, carbohydrates, proteins and DNA chain are destroyed and microorganism is not able to stay alive anymore [40]. Additionally, Sawai and colleagues [41] confirmed that in the case of bulk zinc oxide nanoparticles, the external formation of hydrogen peroxide is the reason of their antibacterial activity. Concerning the biocidal properties, the releasing of zinc ions is also a very important issue. Thanks to the fact that zinc oxide has an amphoteric nature it is able to react with both acids and alkalies. As a result the zinc ions, Zn^2+^ are formed. The released ions are immediately attached to the biomolecules and the growth of microorganism is inhibited. It has been confirmed that the toxicity of ionic form of metals is few times higher than their oxide form. It is directly related to their increased bioavailability [40]. The measurement of antimicrobial activity of obtained compositions was performed on agar dishes. It is known that the migration rate of zinc oxide particles in such a medium is lower than in the case of zinc ions. Moreover, considering the pH of obtained compositions which had the slightly acidic nature, it may be assumed that the mechanism of destroying the studied strain was based on zinc ions releasing, just like in the studies performed by Pasquet and colleagues [42].

#### 3.2.3. Statistical analysis

**Figure 6.**
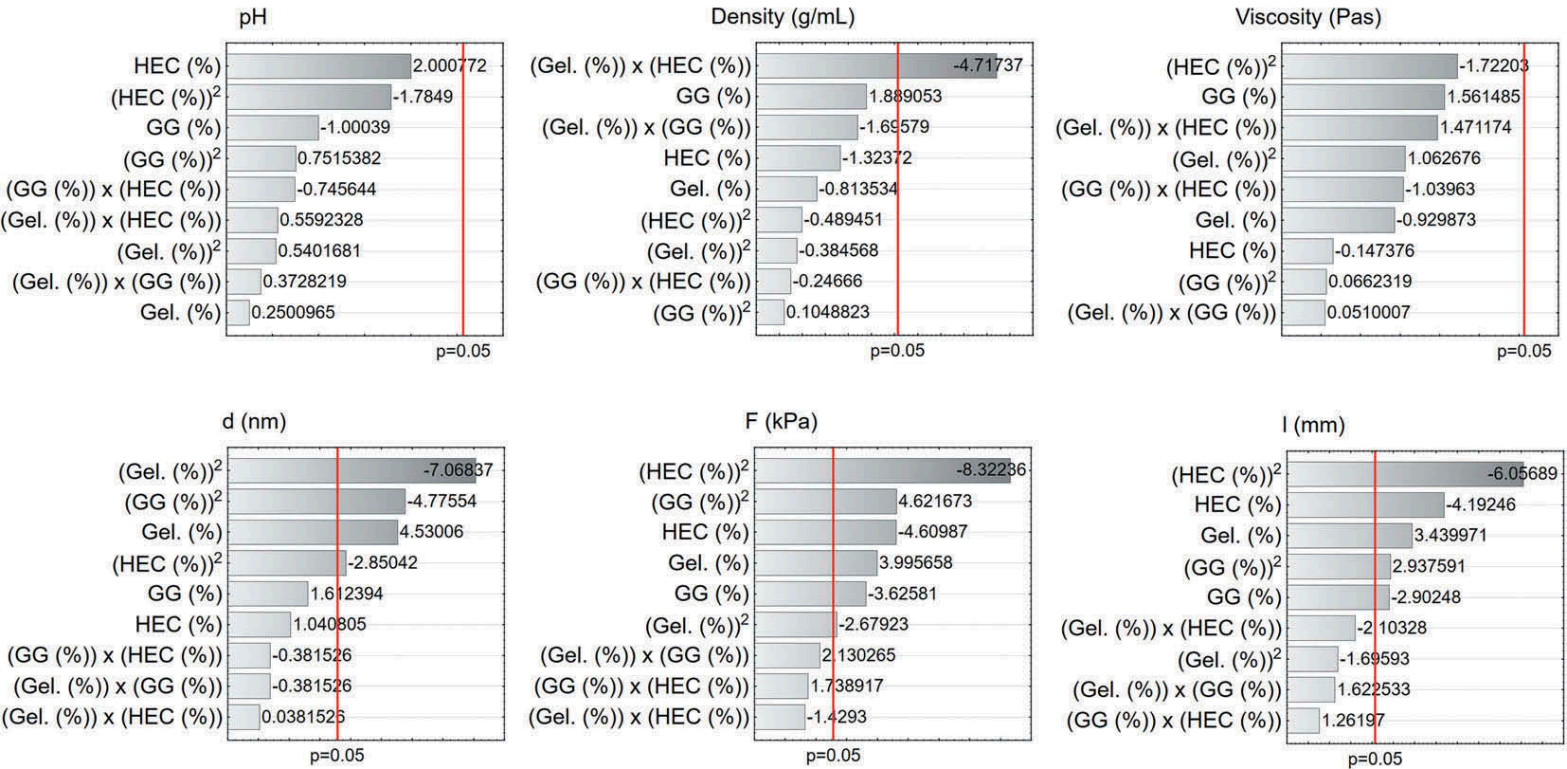
Pareto charts for prepared compositions.

Whether input parameters have a significant influence on output parameters was checked. The input parameters were the concentrations of gelatine (Gel. (%)), guar gum (GG (%)), and hydroxyethyl cellulose (HEC (%)). The group of output parameters consisted of pH, density (kg/m^3^), viscosity (kg/m·s), size of zinc oxide nanoparticles (d (nm)), maximum bond strength (F (kg/m·s^2^)), and absolute elongation (l (m)). Figure 6 presents the Pareto charts for all parameters. Parameters that are statistically significant for the established p value (p < 0.05) are denoted with a red line. Neither pH nor viscosity depends on any input parameter. It is possible that other components in the formulation, such as plasticizers, had a bigger impact on the viscosity of the product, given their higher concentration in the compositions. The product of gelatine and hydroxyethyl cellulose concentrations is the parameter that affects the density with a statistical significance. The size of zinc oxide particles was dependent on gelatine concentration in a linear and quadratic function and both guar gum and hydroxyethyl cellulose concentrations in a linear function. All input parameters in both linear and quadratic functions influence the values of maximum bond strength. The absolute elongation is dependent on all parameters in both linear and quadratic functions excluding gelatine concentration in quadratic function.

**Figure 7.**
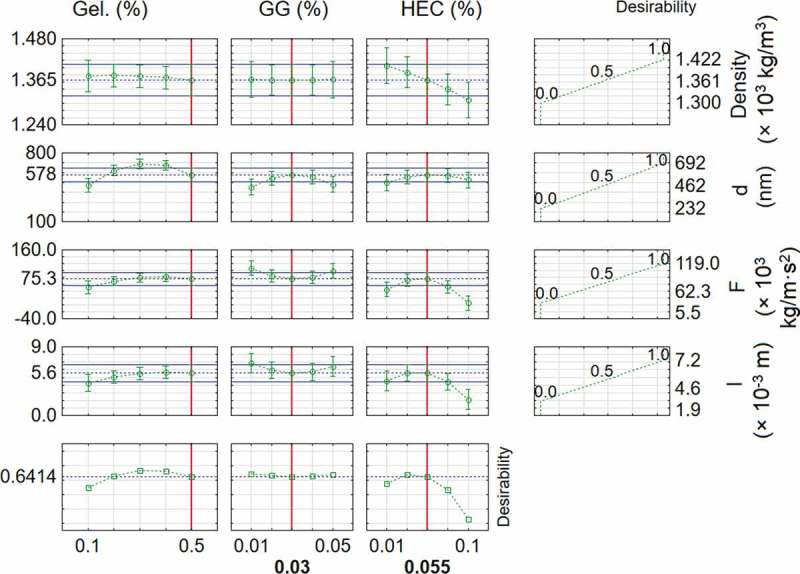
Approximation profiles for prepared compositions.

The approximation profiles are presented in Figure 7. Based on an assessment of the approximation profiles, the influence of independent parameters on dependent variables and the determination of the specific values of input parameters ensure that reaching the desired values of the output parameters is possible. Higher values of density are more desirable. This results from the desire to obtain the right consistency that would ensure application of the product in a proper way so that it would not be detached from the surface and not run down. A smaller size for the zinc oxide particles is more desirable: the smaller the size of the nanoparticles, the greater the share of active atoms located on the particle’s surface. Higher values of both maximum bond strength and absolute elongation are more desirable. The product should adhere firmly to the surface and it should be flexible so as not to be broken. In order to obtain satisfactory bio-adhesion between the composition and the surface to be treated, high bond strengths are required. After solidification, it is desired that the films will be easily peeled off from the treated surface but without tearing due to weak internal bonds.

Neither gelatine nor guar gum significantly influences the density. Increasing the concentration of hydroxyethyl cellulose results in lower density for the compositions. Taking into consideration the influence of input parameters on the size of zinc oxide particles, there are an inflection points. In all cases, initially when the concentrations increase, the size increases as well. After the gelatine concentration reaches a value of 0.3%, the size starts to decrease. The inflection point for guar gum concentration is equal to 0.03% and for hydroxyethyl cellulose it is 0.055%. When the gelatine concentration increases, the maximum bond strength increases as well. However, after reaching 0.4%, its value slightly decreases. The lowest value of guar gum concentration gives the highest maximum bond strength. Neither low nor high values of hydroxyethyl cellulose concentration favour the efficient maximum bond strength. The optimal hydroxyethyl cellulose concentration is equal to 0.055%. The absolute elongation increases when the gelatine concentration increases. Both the lowest and the highest guar gum concentrations give desired values of absolute elongation. However, considering the pro-ecological and economical arguments, the lowest value is better. When the hydroxyethyl cellulose concentration increases, the absolute elongation initially increases as well, however after reaching an inflection point it begins to decay.

With the approximation profiles in relation to properties desirability, it is possible to conclude that the best formulation would be the composition prepared with the highest concentration of gelatine, the lowest concentration of guar gum, and the average concentration of hydroxyethyl cellulose. The closest sample to that case is composition L10, which has the maximum concentration of gelatine and average content of both guar gum and hydroxyethyl cellulose. Sample L10 presents higher density, maximum bond strength and absolute elongation in relation to the average of the all compositions. The zinc oxide particle size is above average; however, the activity of the particles was confirmed in microbiological analysis, so it is highly acceptable.

## 4. Discussion

Zinc oxide has been studied for its antimicrobial properties since the decade of 1950. Like other oxides, such as calcium and magnesium, ZnO presents microbicide activity against bacterial strains. Factors like surface area and charge, size, shape, and chemical reactivity of the nanoparticles influence the antimicrobial mechanism and activity of ZnO [43]. Among these, the most important parameter is the size of the nanoparticles (NPs) [44]. At nanoscale, ZnO particle size is comparable to that of biological molecules like living cells. This allows them to access their internal parts and affect cellular metabolism. The main mechanism responsible for the cytotoxic effect in microbial cells is the generation of reactive oxygen species (ROS) by the ZnO NPs [45]. The mechanism can be explained because of the semiconducting properties of ZnO. Exciting electrons from the valence band to the conduction band and therefore generating positive holes leads to the occurrence of hydroxyl radicals. Even in the lack of external energetic stimulus, the defects in the crystals of ZnO can create electron-hole pairs and have the same effect. The excess of ROS around the cells leads to cellular death due to DNA damage, imbalances in proteins and lipids, and alterations in the cell cycle related to oxidative stress [9,45]. When it comes to selectivity, studies show that ZnO presents antimicrobial activity against both Gram-positive and Gram-negative bacteria, being more effective in the presence of the second group. The difference in action is due to the amount of intracellular antioxidants found in each group and also to the polarity of the membranes. Gram-negative bacteria have more negative cell membranes and a lower concentration of antioxidants; this allows the antibacterial mechanism of ZnO to be more effective [46]. If combined with other antibacterial agent, zinc oxide improves the activity of the system, as in the case studied by Bhadra et al. [47] where ZnO nanorods were encapsulated in a chitosan matrix. The ZnO-chitosan composite presented better antibacterial action against *E. coli* than each of the components in isolation and also when compared to amoxicillin. In practical applications, nanoparticles are often found combined with other materials such as polymers. According to Ahangar et al. [48], the addition of nanoparticles of ZnO to different polymeric matrixes had a positive impact on the mechanical, electrical, optical, and antimicrobial properties of the resulting films.

PVA is a synthetic polymer obtained through the hydrolysis of polyvinyl acetate. It is biodegradable, water-soluble, hydrophilic, non-toxic, and biocompatible. PVA also presents good film forming and mechanical properties [49,50]. Films of PVA can be obtained through a solution casting technique. Due to its biodegradability, PVA is commonly applied in blends with biopolymers such as cellulose derivatives and gelatine for obtaining flexible films [51–53].

Other researchers have studied the preparation of similar materials. Mishra and colleagues [54] obtained PVA coatings with embedded silver nanoparticles. PVA played the role of nanoparticles stabilizing agent. The synthesis was assisted by microwaves, which shortened the process time. The material also included chitosan. It was turn out that the releasing of silver ions has a long-term character. Thanks to that it is possible to ensure the long-lasting antibacterial properties. What is more, the enrichment of coatings with silver nanoparticles led to enhance their mechanical properties comparing to coatings consisted of PVA and chitosan only.

Bhowmick and Koul [55] developed a novel PVA-based hydrogel scaffold containing silver nanoparticles. Authors claim that thanks to the fact that the product is able to absorb a significant amount of water, it is possible to apply it as a wound-healing hydrogel material. Silver nanoparticles are released from the product for at least 96 h and thus the antimicrobial environment is ensured for this period. It was found that the minimal inhibiting concentration against Gram-positive and Gram-negative bacteria strains was equal to 7.81 × 10^−6^ and 3.90 × 10^−6^ kg/kg, respectively. In order to improve the film-forming properties of PVA, natural stabilizing agents were added to the formulations. When hydrated, stabilizing agents form a three-dimensional and crosslinked structure, acting as thickeners, and improve the film-forming, mechanical, and physical properties of the compositions. They also form films around the suspended solid particles. This prevents agglomeration and sedimentation and reduces leaching from the composition into other media [12]. Stabilizing agents are also called hydrocolloids, which are a set of proteins and polysaccharides from natural, semisynthetic, and synthetic sources. Even though their concentration in formulations is usually under 1%, hydrocolloids have a great influence on properties like rheology, texture, heat and shear stability [56]. In the prepared compositions, three natural stabilizing agents were used in combination: gelatine, guar gum, and hydroxyethyl cellulose.In order to increase the affinity between the resulting composition and the microbiological biofilm present on the treated surface, it is necessary to add substances for achieving the effect of ‘engagement’, or bio-adhesion. The adhesion of microbial film to any surface is affected by extracellular polymers which are produced by microorganisms. It concerns mainly lipopolysaccharides and proteins, among others. The enrichment of a composition with compounds such as casein (protein), sucrose (carbohydrate), or chitosan (polysaccharide) could have increased the adhesion of the biofilm to the applied material [57].

The adhesion of microbial cells to the applied coating is a complex process and consists of many stages. Extracellular polymeric substances that are extracted by microbial cell are able to trap ions and both the inorganic and organic nanoparticles. This extracellular matrix has viscoelastic nature and the polymeric substances are located mainly at its surface fringes. Thus they physically catch the nanoparticles of applied coatings. Many factors may influence on the bonding effectivity. The group of most affecting factors includes i.a. pH, ions concentration, types of ligands, the values of both hydrodynamic and thermodynamic forces (Brownian motion) and gravity. When the distance between microbial cells and coating surface is less than 1.5 × 10^−9^ m, then specific bonds may be formed. These are i.a. hydrogen bonds, ion bonds, covalent carbon-carbon bonds and van der Waals forces [58]. Van der Waals forces most strongly determine the effective adhesion of microbial cells to the structure of the applied coating [59,60].

The stronger adhesion of applied coating to the microbial biofilm is ensured by the presence of substances such as casein, sucrose, or chitosan in the composition structure. The interaction between these substances and extracellular polymers that are produced by microbial biofilm enhances the ‘engagement’ effect.

## 5. Conclusions

The performed studies had the objective of developing and characterizing antimicrobial compositions based on PVA and ZnO. Zinc oxide nanoparticles were successfully prepared. Characterization by DLS, XRD, and SEM-EDX indicated satisfactory size (given the absence of stabilizing agents in the preparation method), homogeneous distribution of shape and size, as well as good purity and crystallinity of the particles. Microbiological analysis indicated that the ZnO present in the formulations had effective microbicide action against *Aspergillus niger*.

The developed compositions presented excellent film-forming and physicochemical properties, fitting the practical application for which they were designed. The compositions presented low transdermal diffusion, demonstrating stability of the suspensions. The strong adhesion forces and microbiological activity would result in satisfactory microbial treatment of surfaces.

The effect of gelatine, guar gum, and hydroxyethyl cellulose on the properties of the compositions was evaluated through statistical analysis. Even though the concentration of stabilizers was not high enough to influence some parameters, such as viscosity and ZnO particle size, they were found to have significant influence on the mechanical and physical properties.

Composition L10 was chosen as the one best fitting the desired physicochemical properties and antimicrobial activity. The concentrations of the additives in L10 were as follows: 0.5% gelatine, 0.03% guar gum, and 0.055% hydroxyethyl cellulose. Composition L10 is a base for further development and upscaling.

## Highlights

- PVA-based compositions were prepared with zinc oxide nanoparticles added.
- The influence of functional agents was examined.
- The antimicrobial properties of the compositions were tested against *Aspergillus niger* .
- Zinc oxide nanoparticles did not penetrate the transdermal diffusion membrane.
